# Evaluation of the Effects of STZ-Induced Diabetes on *In Vitro* Fertilization and Early Embryogenesis Processes

**DOI:** 10.1155/2013/603813

**Published:** 2013-03-24

**Authors:** Hüseyin Aktuğ, Vildan Bozok Çetintaş, Ayşegül Uysal, Fatih Oltulu, Altuğ Yavaşoğlu, Saadet Özen Akarca, Buket Kosova

**Affiliations:** ^1^Department of Histology and Embryology, Ege University Medical School, 35100 Izmir, Turkey; ^2^Department of Medical Biology, Ege University Medical School, 35100 Izmir, Turkey

## Abstract

The aim of this study was to investigate the effects of experimentally induced diabetes on (a) germ cells, (b) *in vitro* fertilization (IVF) success rate, and (c) gap junction and cell adhesion molecule gene and protein expressions during the early blastocyst period. Germ cells were obtained from healthy and diabetic rats, analyzed for number, motility, and morphology, and used for IVF. After reaching the early blastocyst stage, the expressions of genes encoding gap junction proteins and cell adhesion molecules were analyzed by quantitative RT-PCR. Histomorphologically and immunohistochemically analyses were also performed. Diabetes significantly affected sperm number and motility and the development of oocytes. Gene expressions of **β**-catenin and connexin family members and protein expressions of E-cadherin and connexin-43 significantly decreased in groups including germ cells isolated from diabetic rats. Connective tissue growth factor expression increased in groups that included sperm cells isolated from diabetic male rats, whereas mucin-1 expression increased in the group that included oocytes isolated from diabetic female rats paired with sperm cells isolated from healthy male rats. In summary, experimentally induced diabetes was found to influence gap junctions, cell adhesion molecules, and associated proteins which all have important roles in germ cell maturation, fertilization, and development.

## 1. Introduction

Diabetes is one of the leading causes of mortality and morbidity among chronic diseases across the world. A number of diabetes-related outcomes, like oxidative stress, also lead to infertility problems among men and women. Diabetic injury on ovarian and testicular tissues highly effects the growth of oocyte and sperm germ cells (oogenesis/spermatogenesis) [1–3]. Changes in mRNA or protein synthesis and expression of maternal origin or paternal influences such as reduction in testicular weight, abnormal spermatogenesis, sperm number, and motility were the most frequently encountered problems [4–6]. Usually, fertilization and preimplantation periods consist of many critical and unique processes including zygotic transcription, first-cell differentiation and specific cell-cell adhesion [7, 8]. Therefore, it is very important during this period to investigate the effects of diabetic injury on germ cells, especially at the molecular level on connection complexes and cell adhesion proteins. 

Gap junction proteins of the connexin (Cx) gene family not only build channels between cells through which low-molecular-weight molecules and second messengers can be shared, but also facilitate homeostatic and developmental processes in the cells. Cx43 has been shown to play a critical role in embryonic development, contraction of the excited cells, tissue homeostasis, normal cell growth, and differentiation [9]. Additionally, cadherin cell adhesion molecules have significant roles with regard to cell migration, polarity, and sorting throughout morphogenesis. Not only is the cadherin family member E-cadherin responsible for compaction of the embryo, but it is also a key organizer of adhesion junctions in epithelial tissue [10–12]. Cadherins which are located at the plasma membrane are intracellularly connected to the actin cytoskeleton via special proteins like catenins [13]. Gap junction and cell adhesion proteins are also associated with each other and exhibit a functional cooperation to increase the stability of cell-cell and cell-extracellular matrix structures [14]. When putting it all together it seems clear that the mutual cooperation of gap junctions, cellular adhesion molecules, and intracellular cytoskeleton proteins are necessarily required for the ordered progression of the embryonic period.

In this study, we aimed to investigate the reflections of this cooperation during early embryogenesis by using an experimental rat diabetes model to determine the effects of diabetic injury in germ cells and to evaluate gender-dependent (sperm/oocyte) consequences regarding the early blastocyst period. By revealing the problems that can occur in the germ cells of diabetic animals during early embryogenesis, new cellular treatments can be designed for resolving diabetes-related infertility.

## 2. Materials and Methods

### 2.1. Animals and Experimental Design

The study protocol complied with the European Community Guidelines for the use of experimental animals. All experiments were approved by the local Animal Ethics Committee at Ege University School of Medicine. In total, one hundred ten *Rattus albinus *male and female rats weighing 180–320 g were selected for this study, housed under standard laboratory conditions (12 : 12 h light : dark, 22°C room temperature, ~60% humidity), and studied in four different groups: Group 1, healthy male/healthy female (10 males/10 females, *n* = 20), animals in this group have not been subjected to any drugs or surgical interventions and served as controls; Group 2, healthy male/diabetic female (15 males/15 females, *n* = 30); Group 3, diabetic male/healthy female (15 males/15 females, *n* = 30); Group 4, diabetic male/diabetic female (15 males/15 females, *n* = 30). Diabetes was induced by a single intraperitoneal injection of STZ (Sigma Chemical Co., St. Louis, MO, USA) 50 mg/kg body weight dissolved in 0.1 mol/L sodium citrate buffer (pH 4.7). A 5% dextrose was administered for the first 24 h. Fourty-eight hours after administration of STZ, the tail vein blood glucose level was measured in all animals. Blood glucose levels of 250 mg/dL and above were considered diabetic due to clinical observations (polyuria and polydipsia) and laboratory findings. Scarification of all rats was initiated with ketamine 50 mg/kg body weight by day 15.

### 2.2. Sperm Collection

Male rats that did at least not mate 3 weeks before the beginning of the study were selected, and after scarification adipose tissue and blood vessels were carefully removed from the cauda epididymis and vas deferens which were dissected under an inverted microscope. Samples were then put on Ham's media and the surface of the cauda epididymis was cut with a 0.5 mL insulin syringe. Semen was released with the help of a forceps that was pushed over the vas deferens that was collected into conical tubes.

*Swim-Up Procedure.* This analysis was performed to collect and clean the motile sperms from cellular debris, and to enrich the sperm population in motile cells. Semen in Ham's media was centrifuged until two layers were visible. Then the tube was tilted by 45° to yield a maximum surface area for the sperms to swim up and be incubated for 1 h (37°C, 5% CO_2_). Sperm motility and number were evaluated with a Makler counting chamber before and after swim up.

*Histomorphologically Analyses of Sperms.* Before the swim-up analyses, sperms were histomorphologically analyzed after semen samples were spread onto microscope slides and stained after the diff-quick method (hematoxylin and eosin; H&E) as described previously [15].

### 2.3. Oocyte Collection

First, 10 IU of pregnant mare serum gonadotropin (PMSG) and then 36 h later human chorionic gonadotropin (HCG) were injected into ~3-months-old female rats. After waiting for another 36 h, oocytes were collection into HEPES buffer solution (Biological Industries Israel Beit-Haemek Ltd.). This oocyte pick-up (OPU) procedure was performed under a dissection microscope (Olympus SZ61).

*Histomorphologically Analyses of Oocytes.* Oocyte morphology is very important for successful *in vitro* fertilization, and oocyte maturation grades affect insemination time. Therefore, oocytes were evaluated according to the following grading system during OPU: Grade 1 mature oocytes, regular cumulus cells, corona radiata with radial diffusivity, prominent zona pellucida, and clear cytoplasm; Grade 2 immature oocytes, dark and compact cumulus cells, densely and close corona cells, no prominent zona pellucida, and lost transparency of cytoplasm; Grade 3 postmature oocytes, dense cumulus cells, irregular but visible zona pellucida, and dark and granular cytoplasm; Grade 4 atretic oocytes, very few cumulus cells, irregular corona radiate, and ooplasm. This grading system was used for oocytes collected from 25 control and 24 diabetic female rats (6 of total 30 diabetic female rats died before oocytes collection).

### 2.4. *In Vitro* Fertilization Procedure

Oocytes were collected and transferred into four-well culture dishes (Nunc, Roskilde, Denmark) containing 1 mL of Single Step Medium (Irvine Scientific, USA), sealed with mineral oil and left for incubation (37°C, 5% CO_2_). After ~4–6 h of incubation, maturation was achieved. Sperms were first evaluated for number and motility, and then left 45 min for incubation (37°C, 5% CO_2_) before insemination. For the fertilization process, oocytes were transferred with transfer pipettes into Gamete, Fertilization and Embryo Culture Medium (SSM, Irvine Scientific, Santa Ana, CA, USA) containing culture dishes together with 50,000–100,000 motile sperms/mL per oocyte. After 1 day of insemination, fertilization was evaluated by the presence and number of pronuclei (PN). Fertilized oocytes having 2 PN were transferred into new culture dishes and followed up and maintained until the early blastocyst period.

### 2.5. Embryo Blocking

Embryos that reached the early blastocyst period were fixed in 4% paraformaldehyde (Sigma Chemical Co., St. Louis, MO, USA) containing phosphate buffered saline solution (PBS, pH 7.4) for 45 min at 4°C [16]. After fixation, these embryos were washed several times with PBS, dehydrated through a graded ethanol series (80%, 95%, and 100%, sequentially), cleared in xylene, and embedded in paraffin.

### 2.6. Immunohistochemical Staining

Two *μ*m thick cross-sections were taken with a microtome (Leica MR 2145) from paraformaldehyde-fixed paraffin-embedded (PFPE) blastocysts, floated in a sterile bath, picked up onto poly-L-Lysine-coated glass slides, and dried at room temperature (RT). After overnight incubation at 60°C, they were dewaxed in xylene for 30 min, rehydrated through a graded ethanol series (100%, 95%, 80%, and 70%, sequentially), washed in distilled-H_2_O and PBS for 10 min, treated with 2% trypsin containing 50 mM Tris buffer (pH 7.5) at 37°C for 15 min, and then washed again with PBS. Sections were delineated with a Dako pen (Dako, Glostrup, Denmark), incubated in 3% H_2_O_2_ solution for 15 min to inhibit endogenous peroxidase activity, and washed with PBS. Primary antibodies mouse anti-Cx43 (1 : 100 dilution; Santa Cruz, CA, USA) and rabbit anti-E-cadherin (1 : 100 dilution; Santa Cruz, CA, USA) were applied in an incubator at 57°C and washed with PBS. Afterwards, the biotinylated secondary IgG antibody was applied and washed with PBS before incubating with the streptavidin-peroxidase conjugate (Histostain Plus, Invitrogen, Camarillo, CA, USA) and 3,3-diaminobenzidine tetrahydrochloride (DAB; Invitrogen, Camarillo, CA, USA) for 5 min to visualize immunostaining. The whole procedure was finished after counterstaining the sections with Mayer's hematoxylin (Sigma Chemical Co., St. Louis, MO, USA).

### 2.7. Quantitative Reverse Transcriptase-Polymerase Chain Reaction

RNA isolation from blastocysts was performed with the Micro-FastTrack kit (Invitrogen, Camarillo, CA, USA) and 10 *μ*g RNA was reverse transcribed with the high fidelity Transcriptor First Strand cDNA Synthesis kit (Roche Applied Science, Basel, Switzerland). A custom array panel (Roche Applied Science, Basel, Switzerland) was designed for the quantification of 24 target genes and 3 housekeeping genes (*H6pdh*: hexose-6-phosphate dehydrogenase, *RGD1560437*: similar glyceraldehyde 3 phosphate dehydrogenase and Actb: **β*-actin*) by reverse transcriptase-polymerase chain reaction (RT-PCR) using the LightCycler 480 instrument (Table 1). **β*-actin* was expressed in all samples (probably, because the other two housekeeping genes are not as strongly expressed at this early stage of development) and used for calculating the relative quantification of all genes with the instrument's own software. Nevertheless, the expression of a fourth housekeeping gene (ribosomal RNA, *Rrp1*) was confirmed in an extra PCR reaction, but not used for calculations.

### 2.8. Statistical Analyses

For statistical analyses of sperm numbers and oocyte qualities the SPSS 13.0 software for Windows was used. Statistical analyses of gene expressions were calculated with the LightCycler 480 software. After normalization to the **β*-actin* housekeeping gene, fold change analysis was performed for the expression values and *P* values were calculated.

## 3. Results

### 3.1. Semen Analyses

**Figure 1:**
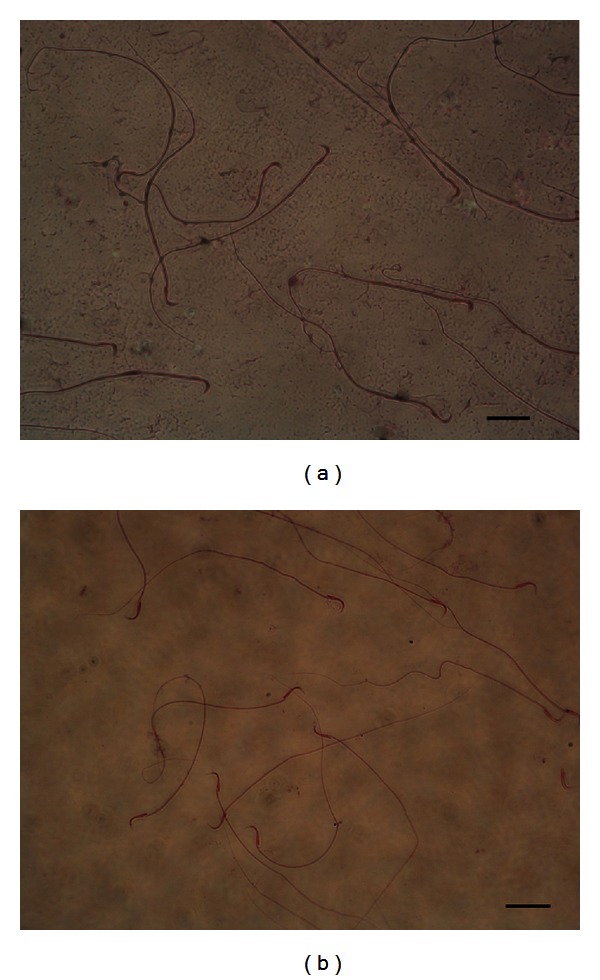
Diff-quick staining of sperm cells. (a) Sperm cells isolated from healthy male rats; (b) sperm cells isolated from healthy male rats; H&E staining, magnification 40x, scale bar: 125 *μ*m.

Semen samples from ten randomly selected male rats (*n* = 5 control; *n* = 5 diabetic) were collected from the cauda epididymis and counted with a Makler counting chamber before and after swimup. Mean sperm numbers were found to be 1.5 × 10^7^ and 6 × 10^6^ in the control and diabetic groups, respectively. This result clearly shows the significant reduction in sperm number in the diabetic group when compared with the control group (*P* = 0.032). However, when diff-quick staining was performed to assess possible sperm abnormalities, no differences in sperm tail, head, and neck morphology could be observed in both groups (Figure 1). Swim-up analyses of sperms in both groups was also performed before *in vitro* fertilization (IVF) (Table 2).

### 3.2. Oocyte Analyses

**Figure 2:**
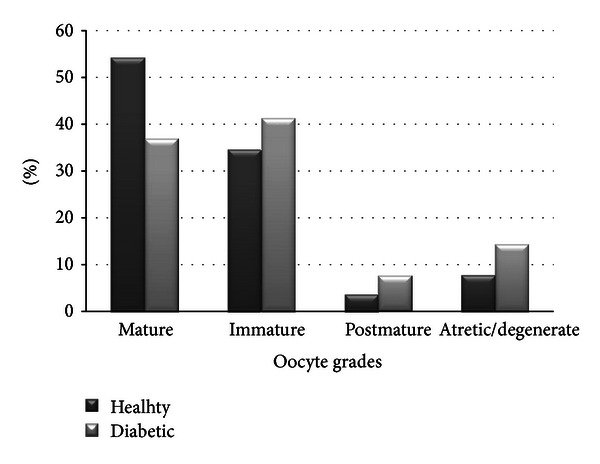
Comparison of oocyte qualities between healthy and diabetic female rats.

Oocytes were collected from forty-nine female rats (*n* = 25 control; *n* = 24 diabetic) under a dissection microscope. A total of 615 oocytes (336 = control; 279 = diabetic) were evaluated and oocyte qualities were determined as mature 54.2%, immature 34.5%, postmature 3.6%, and atretic 7.7% in the control group; and mature 36.9%, immature 41.2%, postmature 7.5%, and atretic 14.3% in diabetic group (Figure 2). The differences of mature, immature, postmature, and atretic oocytes between the control and diabetic groups were found to be statistically significant (*P* < 0.001); that is, percentage of mature oocytes was lower, whereas percentage of postmature and atretic oocytes was higher in the diabetic group. 

### 3.3. *In Vitro* Fertilization

After 6 h of incubation for immature oocyte maturation, a total of 285 mature (182 = control; 103 = diabetic) and 231 immature (116 = control; 115 = diabetic) oocytes were collected for the IVF procedure. These oocytes were divided into four different groups and 50.000–100.000 of motile sperm/mL were added (Table 3). After 24 h of insemination, fertilization was evaluated by the observation of various pronuclear forms. IVF outcomes were statistically significantly different between both groups (*P* < 0.001; Table 3). Whereas the highest IVF success rate was obtained for the healthy male/healthy female group (53.3%), the lowest rate was obtained for the diabetic male/diabetic female group (22.8%). Others were the diabetic male/healthy female group with an IVF success rate of 45.3% and the healthy male/diabetic female group with an IVF success rate of 28.7%. On the other hand, reaching the early blastocyst stage of IVF was nearly 2-fold higher in the healthy male/healthy female group than that in the other 3 groups, which included at least one type of diabetic germ cells. Blastocyst samples were collected at the 5th day of IVF for further immunohistochemical analyzes and mRNA isolation.

### 3.4. Immunohistochemical Analyses

**Figure 3:**
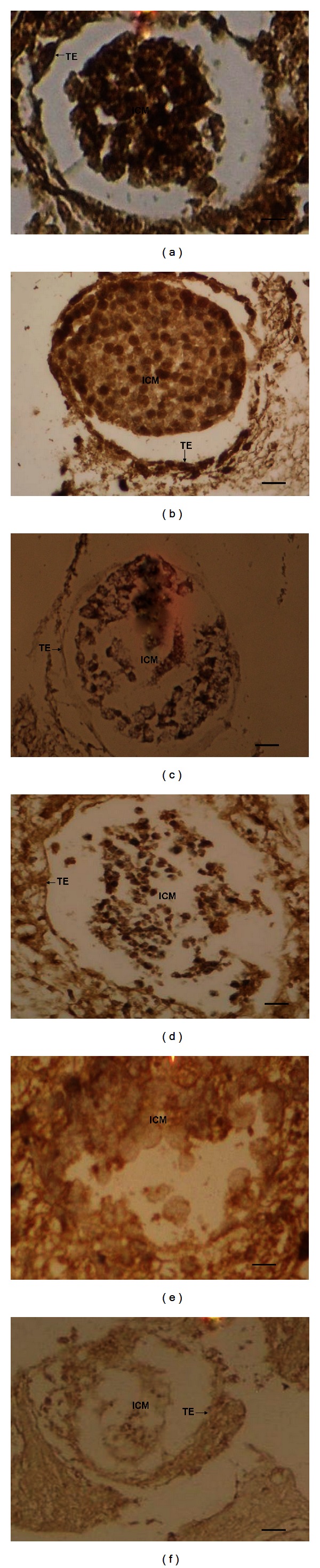
E-cadherin and Connexin 43 protein expressions. (a) Group 1, healthy male/healthy female, E-cadherin, magnification 20x*; (b) Group 2, healthy male/diabetic female, E-cadherin, magnification 100x**; (c) Group 3, diabetic male/healthy female, E-cadherin, magnification 40x***; (d) Group 4, diabetic male/diabetic female, E-cadherin, magnification 100x**; (e) Group 1, healthy male/healthy female, Connexin 43, magnification 100x**; (f) Group 4, diabetic male/diabetic female, Connexin 43, (diabetic female/male, magnification 40x***. ICM: inner cell mass; TE: trophectoderm; scale bars: *250 *μ*m, **50 *μ*m, and ***125 *μ*m.

IVF embryos that reached the blastocyst stage were embedded in paraffin and analyzed for E-cadherin and Cx43 protein expressions. E-cadherin expression was found to be similarly higher in groups that included germ cells from healthy males (Groups 1 and 2) than in the groups that included germ cells from diabetic males (Groups 3 and 4; Figures 3(a)–3(d)). Group 4 displayed the lowest level of E-cadherin immunoexpression. When the expression of Cx43 was analyzed, it was found to be the highest in the group where germ cells from healthy male/healthy females were matched (Group 1; Figures 3(e)-3(f)). Although, the expression of Cx43 was lower in the other groups (Groups 2, 3, and 4), no significant expressional differences could be detected between them.

### 3.5. Gene Expression Analyses

**Figure 4:**
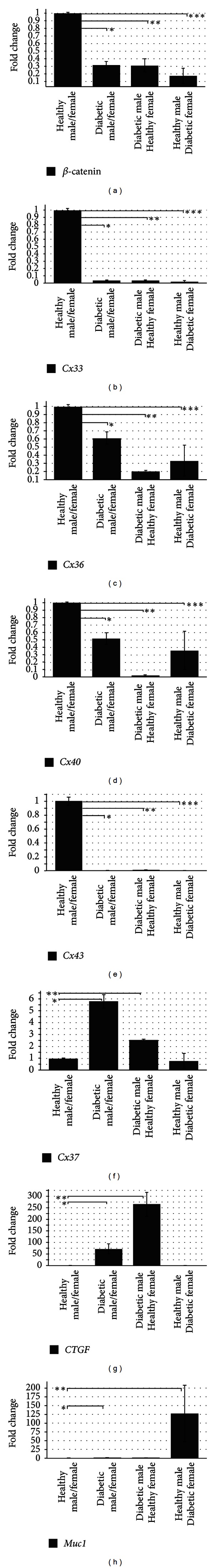
Significantly increased or decreased fold regulations of gene expression levels. (a) *β*-catenin (*,  **,  ***P < 0.0001); (b) Connexin 33 (*,  **,  ***P < 0.0001); (c) Connexin 36 (*,  **P < 0.0001; ***P = 0.004); (d) Connexin 40 (*,  **P < 0.0001, ***P = 0.01); (e) Connexin 43 (*,  **,  ***P < 0.0001); (f) Connexin 37 (*,  **P < 0.0001); (g) CTGF (*,  **P < 0.0001); (h) Muc1 (*P = 0.026, **P = 0.022).

Gene expression analyses revealed that **β*-catenin* and most of the connexin gene family members were decreased in all diabetic germ cell including IVF groups (Groups 2, 3 and 4; Figures 4(a)–4(e)). However, *E-cadherin* gene expressions were not different between the groups. Interestingly, the connective tissue growth factor (*CTGF*) and *Cx37* gene expression levels were increased in the groups that included germ cells isolated from diabetic males (Groups 3 and 4; Figures 4(e)-4(f)). In addition, only the expression of *Mucin-1* (Muc1) that encodes a membrane-bound protein was increased in the healthy male/diabetic female group (Group 2; Figure 4(g)).

## 4. Discussion

It is assumed that the effects of diabetes on zygotic transcription in spermatozoa are important in the development of infertility in men. Depending on the present paternal metabolic abnormality, sperm cell division or development can be affected [17]. In this study, number and motility of sperm cells isolated from diabetic males were found to be significantly decreased when compared with sperms from healthy controls. Nevertheless, when sperm morphology was evaluated using Kruger's strict criteria, no light microscopically visible significant differences could be detected between both groups. There might be differences in the distribution of nuclear chromatin but these can only be evaluated ultrastructurally by electron microscopy.

Generally, the swim-up method is used for the selection of good quality spermatozoa to improve embryo quality and pregnancy rates and to reduce the number of miscarriages after an IVF attempt [18]. Usually, the motile sperm population selected by this method includes increased rates of morphologically normal and motile sperms and decreased rates of internal abnormal (e.g., damaged DNA, reduced chromatin condensation, and apoptotic) spermatozoa [19]. By using the swim-up method, we could also increase the number of sperms with high motility grades obtained from the control and diabetic groups.

Maternal diabetes is known to affect embryos during the preimplantation period [20] and oocyte meiotic progression [5, 21, 22]. Therefore, the best way to improve embryo quality is to improve oocyte quality. It is known that the oocyte cytoplasm plays a very important role in oocyte maturation; that is, if the cytoplasm of a oocyte is insufficiently maturated, it will fail to promote male pronuclear formation and increase the rate of chromosomal abnormalities after fertilization [23]. Uptake of glucose strongly appears to be mediated by the gap junctional communication pathway that metabolically couples the oocyte with the somatic compartment of the follicle [21, 24]. What we noticed during the oocyte pick-up (OPU) step was how much damage experimental diabetes does generate on the morphology and maturation process of oocytes. Because, in oocytes obtained from diabetic rats, atresia and degenerative structures were found to be more frequent than in oocytes obtained from healthy controls, hence it might be one cause of maternal infertility.

Fertility is directly linked to glucose metabolism as glucose is required for sperm fusion to zona-free murine oocytes [17]. Hyperglycemic conditions and glucose metabolites can be regarded as very important factors for infertility. Indeed, our results verified this point of view, since the overall IVF success rate was found to be higher in the diabetic male/healthy female group when compared with the healthy male/diabetic female group. We also observed that oocytes were generally more stable than sperm cells at the beginning of the fertilization process. But when both types of germ cells were obtained from diabetic rats, IVF success rates dropped substantially.

Smooth progression through early embryogenesis, especially the early blastocyst period, is ensured by two major protein groups: (1) gap junction proteins, and (2) cell adhesion molecules. Gap junction proteins and cell adhesion molecules, as well as tight junctional structures, are all in a dynamic relationship with each other and generate the basis for early embryonic development. Gene expression analysis of Cx gene family members in mice embryos during the preimplantation period has showed that *Cx30, Cx31, Cx36, Cx43, Cx45,* and *Cx57* were predominantly expressed at the two- and four-cell stages, whereas *Cx30.3, Cx31.1,* and *Cx40* were expressed at the eight-cell stage and initially in the cytoplasm [25]. Cell adhesion between blastomeres is known to begin at the eight-cell stage, while the gap junction protein Cx43 moves towards the plasma membrane during the compaction stage [26, 27]. We could identify the expression of *Cx30, Cx31, Cx33, Cx36, Cx37, Cx40,* and *Cx57* in all IVF groups, but particularly *Cx33, Cx36, Cx40, *and* Cx57* expressions were significantly higher in the healthy male/healthy female group. Cx43 is the most dominant connexin expressed during the preimplantation period until the blastocyst period [28]. In an *in vivo* bovine study, it could be also shown that *Cx43* gene expression significantly increased within the progression from the sixteen-cell stage to the blastocyst stage, and that the formed gap junctions were highly essential for cell communication throughout the preimplantation period, particularly in the blastocyst stage [8]. Similarly,  we found that Cx43 was highly expressed at the blastocyst stage, at gene as well as protein level. However, suppressed levels of *Cx43* expression were determined in the groups which included diabetic rat derived germ cells. 

Cadherins are calcium-dependent transmembrane proteins that have important roles in cell adhesion and differentiation. One of its classical members is E-cadherin, localized to lateral cell surfaces and providing continuity to the epithelial layer [11]. During embryogenesis, E-cadherin function is also critical in the compaction process which occurs at the eight-cell stage. Later, differential expression of E- and N-cadherins can drive morphogenesis by stimulating cell aggregation, defining boundaries between cells groups and promoting cell migration [29]. E-cadherin is connected to intracellular actin filaments through anchor proteins like *α*-, *β*-, and *γ*-catenins [14]. In our study, protein expression of E-cadherin was found to be significantly reduced in the groups which included diabetic rat derived germ cells. Additionally, gene expression level of **β*-catenin* was similarly decreased in the same groups. 

CTGF is a critical mediator of extracellular matrix accumulation and diabetic conditions can stimulate its expression [30]. Moreover, an upregulation of the TGF-*β*1/Smad/CTGF signaling pathway was determined in streptozotocin (STZ) induced diabetic rats [31]. In our study, the expression of *CTGF* was significantly increased in the groups that included sperm cells isolated from diabetic male rats and could therefore be interpreted as a positive evidence for the paternal inheritance of *CTGF* effects. 

Muc1 is a highly glycosylated, high molecular weight glycoprotein that is present on epithelial surfaces, including human endometrial epithelial cells [32]. Through its cytoplasmic domain Muc1 directly binds to and stabilize *β*-catenin which, besides its function as an intracellular anchor protein, is a key modulator of several signaling pathways that affect cell motility and morphology [33]. Increased levels of Muc1 at the cell surface can inhibit cell-cell adhesion via steric hindrance of receptor-ligand interactions mediated by cadherin-like adhesion molecules [34]. Although, Muc1 expression in endometrial cells and its regulation during implantation are both well understood, limited information is available concerning its expression level in blastocysts and its possible effects on the successful outcome of the implantation process. But it is known that while Muc1 is initially low expressed in blastocysts, it later specifically increases in the epiblast region [35]. In our study, the expression of *Muc1* was found to be significantly high in the healthy male/diabetic female group. Its high expression may damage cell-cell and/or cell-matrix adhesion, which may in turn impair the proper assembly of intracellular cytoskeletal structures at these sites. All these effects might be gender-based associated, that is, especially important if oocytes are affected due to maternal diabetes.

In conclusion, experimentally induced diabetes was found to influence gap junctions, cell adhesion molecules, and associated proteins which all have important roles in germ cell maturation, fertilization, and development. We believe that more detailed molecular and morphological analyses of these structures in early embryogenesis will help to decipher the mechanisms that lead to diabetic infertility and may help to design new cellular therapies in the future.

## Figures and Tables

**Table 1:** Details of the genes analysed by quantitative RT-PCR.

Gen ID	Symbol	Full name	Chromosome and location
24392	Gja1 (Cx43)	Gap junction protein, alpha-1 (connexin 43)	20q11
25655	Gja4 (Cx37)	Gap junction protein, alpha-4 (connexin 37)	5q36
50563	Gja5 (Cx40)	Gap junction protein, alpha-5 (connexin 40)	2q34
54256	Gja6 (Cx33)	Gap junction protein, alpha-6 (connexin 33)	Xq21
266706	Gja7 (Cx45)	Gap junction protein, alpha 7 (connexin 45)	10
313126	Gja10 (Cx57)	Gap junction protein, alpha-10 (connexin 57)	5q21
29584	Gjb1 (Cx32)	Gap junction protein, beta-1 (connexin 32)	Xq31
394266	Gjb2 (Cx26)	Gap junction protein, beta-2 (connexin 26)	15p12
117055	Gjb4 (Cx30.3)	Gap junction protein, beta-4 (connexin 30.3)	5q36
29586	Gjb5 Cx31.1	Gap junction protein, beta 5 (connexin 31.1)	5q36
50564	Gjd2 (Cx36)	Gap junction protein, delta 2 (connexin 36)	3q35
192248	Cdh13	Cadherin 13 (T-cadherin)	19q12
116777	Cdh3	Cadherin 3, type 1, P-cadherin (placental)	19q12
25409	Cdh6	Cadherin 6 (K-cadherin)	2q16
84408	Cdh8	Cadherin 8	19p13
117048	Cdh17	Cadherin 17	5q13
116808	Pcdh12	Protocadherin-12 (vascular endothelial cadherin 2)	18p11
24571	Muc1	Mucin-1, cell surface associated	2q34
170921	Itga2	Integrin, alpha 2	2q14
84353	Ctnnb1	Catenin (cadherin associated protein), beta1	8q32
64032	Ctgf	Connective tissue growth factor	1p12
307505	Ctnna1	Catenin (cadherin associated protein), alpha 1	18p11
25361	Vcam1	Vascular cell adhesion molecule 1	2q41
83502	Cdh1	Cadherin-1 (E-cadherin)	19q12

**Table 2:** Motility grades of control and diabetic rat sperms before and after swimup.

		Before swimup					After swimup		
Groups	Sample (*N* = 10)	Total sperm number	Motility grades* (%)				Total sperm number	Motility grades* (%)	
			I	II	III	IV		I	II
Healthy	1	6 × 10^6^	30	40	25	5	25 × 10^6^	60	40
	2	18 × 10^6^	25	40	25	10	35 × 10^6^	60	40
	3	15 × 10^6^	20	50	20	10	38 × 10^6^	50	50
	4	22 × 10^6^	20	50	20	10	40 × 10^6^	80	20
	5	14 × 10^6^	20	50	20	10	27 × 10^6^	75	25

Diabetic	1	4 × 10^6^	15	35	40	10	11 × 10^6^	50	50
	2	4 × 10^6^	10	30	30	30	15 × 10^6^	60	40
	3	2 × 10^6^	5	25	50	20	10 × 10^6^	20	80
	4	8 × 10^6^	10	30	40	20	14 × 10^6^	60	40
	5	12 × 10^6^	10	40	20	30	28 × 10^6^	70	30

*Motility grades I: progressive motility (≥25 mM/sec, practically half of the tail of the sperm head up to 5 times, or the sperm move), II: nonlinear motility, III: nonprogressive motility (≥5 mm/second), IV: immotile.

**Table 3:** Comparison of the *in vitro* fertilization success rates between the groups.

Groups	Total oocyte number	Fertilization (−)		Fertilization (+)	
			RowSpanEmpty	RowSpanEmpty	2-cell stage	8-cell stage	Early blastocyst
(1) Healthy male/healthy female	148	69	37	19	23
		46.6%	25.0%	12.8%	15.5%
(2) Healthy male/diabetic female	108	77	11	11	9
		71.3%	10.2%	10.2%	8.3%
(3) Diabetic male/healthy female	150	82	34	20	14
		54.7%	22.7%	13.3%	9.3%
(4) Diabetic male/diabetic female	110	85	10	7	8
		77.3%	9.1%	6.4%	7.3%
